# B-Vitamin Levels in Human Milk among Different Lactation Stages and Areas in China

**DOI:** 10.1371/journal.pone.0133285

**Published:** 2015-07-17

**Authors:** Xiangnan Ren, Zhenyu Yang, Bing Shao, Shi-an Yin, Xiaoguang Yang

**Affiliations:** 1 National Institute for Nutrition and Health, Chinese Center for Disease Control and Prevention, 29 Nan Wei Road, Xicheng District, Beijing, 100050, China; 2 Beijing Key Laboratory of Diagnostic and Traceability Technologies for Food Poisoning, Beijing Research Center for Preventive Medicine, 16 Hepingli Zhongjie, Dongcheng District, Beijing, 100013, China; Oxford University, UNITED KINGDOM

## Abstract

To determine the contents of B-vitamins in human milk in China, we analyzed 1778 human milk samples from the sample bank of the National High Technique R & D Program (863 Projects) which was a cross-sectional survey and covered 6419 human milk samples from healthy lactating mothers who were at different stages of lactation (0–330 days postpartum) in 11 provinces of China. The contents of free forms of six B-vitamins in these human milk samples were analyzed by using UPLC-MS/MS. The median concentrations of free form of 6 B-vitamins in colostrums, transitional milk, 15–180 d mature milk and 181-330 d mature milk were respectively as follows: thiamin 5.0 µg/L, 6.7 µg/L, 21.1 µg/L and 40.7 µg/L; riboflavin 29.3 µg/L, 40.6 µg/L, 33.6 µg/L and 29.6 µg/L; niacin 470.7 µg/L, 661.3 µg/L, 687.0 µg/L and 571.3 µg/L; vitamin B-6 4.6 µg/L, 16.1 µg/L, 62.7 µg/L and 80.7 µg/L; flavin adenine dinucleotide (FAD) 808.7 µg/L, 1162.8 µg/L, 1023.9 µg/L and 1057.2 µg/L; pantothenic acid 1770.9 µg/L, 2626.8 µg/L, 2213.0 µg/L and 1895.5 µg/L. The contents of 6 B-vitamins varied significantly among the different lactation stages and different areas (coastal area vs inland area, rural area vs urban area). The present study indicated that the concentrations of B-vitamins in colostrum were generally much lower than those in transitional milk and mature milk. Further studies are warranted for their roles and significance on B-vitamins in colostrum in nutrition and metabolism of neonates.

## Introduction

Human milk has been recommended as the optimal food for infants and met almost all the nutrient requirements for infants aged 0 to 6 months. Human milk is not only essential for infants’ growth and development, but also provides immunological protection for infants [1, 2]. Generally, the B-vitamins from human milk are the only source for exclusively breastfed infants [3].

Inadequate B-vitamin intakes could lead to vitamin deficiencies, such as growth retardation, anemia, neurological deficits, beriberi, anorexia or some adaptive disability [4–8]. There has been growing interests in B-vitamins levels of human milk in the past 30 years [6, 8–12]. The contents of B-vitamins (thiamine, riboflavin, vitamin B-6, niacin, flavin adenine dinucleotide (FAD) and pantothenic acid) of human milk have been studied in various countries [13–35] with a limited sample size ranging from 5 to 152 samples. Numerous researches showed that the contents of B-vitamins in human milk were significantly different among different lactation stages [13–15, 17, 18, 25]. Maternal dietary intake could affect many B-vitamins levels in human milk [13, 17, 23, 33–36]. Maternal thiamine and riboflavin deficiency was closely associated with the lower thiamine and riboflavin in breast milk [27–31]. Sneed *et al* [35] found that the socioeconomic status was closely related to the dietary intakes of B-vitamins of lactating women. The contents of thiamine, riboflavin and niacin of human milk in rural areas were lower than those in urban areas [22, 23]. The level of B-vitamins in human milk also varied among populations across different countries [16, 32, 36].

However, a great majority of available studies were with small sample size, which might limit the potential to generalize the results to a large population. Additionally, few studies reported the content of flavin adenine dinucleotide (FAD) in human milk [3]. FAD, a coenzyme, is the major form of riboflavin and can improve the adaptability for environment stress during infancy [37]. Moreover, some studies showed that the geographical and regional factors could explain the variations for some nutrient concentration in human milk, but not for B-vitamins [32, 36].

The aim of the study was to determine B-vitamins (thiamine, riboflavin, vitamin B-6, niacin, FAD and pantothenic acid) levels of human milks sampled from a cross-sectional survey in China and the B-vitamin levels were compared among different lactation stages. The relationship between B-vitamins contents and region (coastal vs inland area, rural vs urban area) was also explored.

## Materials and Methods

### Subjects

The study was approved by the ethic committee of the National Institute for Nutrition and Health, China CDC. Written informed consent was obtained from all participants.

Human milk samples (1778) were randomly selected from the sample bank of the National High Technique R & D Program (863 Projects) from 2010–2013, which was a cross-sectional survey and covered 6419 human milk samples from healthy lactating mothers who were at different stages of lactation (0–330 days postpartum). These breastfeeding women were from 20 sites in eleven provinces, municipalities and autonomous regions, namely Beijing, Shanghai, Guangzhou, Heilongjiang, Yunnan, Neimeng, Xinjiang, Guangxi, Gansu, Zhejiang, Shandong, respectively. Mothers aged 20 to 35 years old with full-term, singleton delivery (37 to 42 weeks) were qualified for the study. Mothers who had mastitis, infectious diseases, cardiovascular diseases, metabolic diseases, mental diseases, used tobacco or participated in clinical interventions were excluded from the study.

### Milk sample collection

Human milk samples were collected in a dim light room without direct sunlight exposure. The collected samples were stored and transported in ice box in the field to protect the vitamins from degradation. One full breast was emptied by using a portable automatic breast pump (HNR/X-2108Z, Shantou, Guangdong, China) at 9:00–11:00 am and the milk was collected into a feeding bottle. After gently up-down shaking the milk bottle for ~10 times, the samples were aliquotted into 15 mL centrifuge tubes (about 9–10 mL in each tube) and were stored at -20°C freezer in the field. Then the frozen samples were shipped to the lab in Beijing under the frozen status and were stored at -80°C freezer until analysis.

### Analysis of B-vitamins in human milk

B-vitamins were analyzed by using ultra performance liquid chromatography tandem mass spectrometry (UPLC-MS/MS). The samples were prepared under the dim light on ice. The analyzed method was based on Dr. Hampel’s method with some modification [16]. Breast milk samples were taken out from -80°C freezer and were thawed over night at 4°C. On the following day, after homogenization by Sonicator (MIRIS, Sweden), 10 μL internal standard and 250 μL methanol were added to 50 μL of homogenized breast milk. The samples were mixed for 1 min and centrifuged at 14480 x g for 10 min at 4°C in order to remove solid particles from the supernatant. The supernatant was transferred into amber tubes and evaporated to dryness (nitrogen-stream, room temperature). The residue was reconstituted and mixed for 1 min in 200 μL pure water. The samples were then back-extracted with 200 μL diethyl ether by mixing for 30 seconds and placing them at 4°C for 10 min in order to remove the hydrophobic molecules. The hydrophilic phase was transferred to centrifuge tubes and was centrifuged at 14480 x g for 10 minutes at 4°C. Finally, 100 μL of the clear supernatant was transferred into 150 μL glass insert in a 1.5 mL amber glass vial and analyzed. UPLC HSS T3 column (2.1 × 100 mm, 1.8 μm) (Waters, USA) was used. The mobile phase was a gradient elution of 2.5 mM ammonium formate aqueous solution (mobile phase B) and acetonitrile (mobile phase A) at a flow rate of 0.35 mL/min. The gradient elution process was: 0–2 min, 1% A; 2–4 min, 1–5% A; 4–5 min, 5–20% A; 5–6 min, 20–25% A; 6–7 min, 25–30% A; 7–7.1 min, 30–95% A; 7.1–9 min, 95% A; 9–9.1 min, 95–1% A; 9.1–11 min, 1% A. The injection volume was 10 μL.

The working standard solution of thiamin, riboflavin, nicotinic acid, pyridoxine, pyridoxamine, pyridoxal ranged from 0.5 to 160 ng/mL (0.5, 1, 5, 10, 25, 50, 80, 100, 125 and 160 ng/mL) and the working standards of pantothenic acid, FAD, nicotinamide ranged from 2.5 to 800 ng/mL (2.5, 5, 25, 50, 125, 250, 400, 500, 625 and 800 ng/mL). The R^2^ of standard curve for all standards ranged from 0.990 to 0.999. The limit of detection (LOD) for all vitamins was between 0.05 and 1.50 μg/L.

### Quality control

National institute of standards and technology (NIST) Standard Reference Material (SRM) 1849a Infant/Adult Formula and the pooled breast milk were used for quality control in each analysis. The accuracies of thiamin, riboflavin, nicotinamide, vitamin B-6 and pantothenic acid in NIST samples were 87–116%, 91–112%, 85–115%, 91–115%, 83–110%, respectively. The relative standard deviation (RSD) meant that standard deviation (SD) divided by the mean value. The RSD results of pooled milk of thiamin, riboflavin, nicotinamide, vitamin B-6, FAD and pantothenic acid were 13%, 17%, 24%, 11%, 26%, and 20%, respectively.

### Statistical analysis

Statistical analyses were performed by using the SPSS software (Ver. 19.0) (IBM, USA). The data were expressed as mean±SD and median with interquartile ranges (p25-p75). The contents of B-vitamins in human milk were natural logarithm transformed because their distributions were skewed. The transformed data were used for statistical analysis. The human milk samples were categorized into colostrum (0–7 d), transitional milk (8–14 d), mature milk (15–180 d) and mature milk (181–330 d). To analyze the effects of different stages of lactation, analysis of variance (ANOVA) was used. To study the relationship between rural and urban areas, coastal and inland areas, and impact of different lactation stages, independent sample t-test was used. Differences were considered significant at *p*<0.05.

## Results

### Characteristics of lactating women in different sites

The characteristics of the subjects were shown in Table 1. According to the number of population, 20 survey sites were classified into rural and urban areas by China Health and Nutrition Survey (CHNS). 20 survey sites were classified into coastal and inland areas based on the geographic feature of China. The coastal areas were all urban areas while the inland areas included the rural and urban areas. Ages and body mass index (BMI) of lactating women were lower in rural areas than in urban areas (ages: 25.2 vs 28.0, BMI: 23.0 vs 24.6) and were higher in coastal areas than in inland areas (ages: 28.2 vs 26.1, BMI: 24.2 vs 23.7). Levels of education and household income per capita were significantly lower in rural areas than those in urban areas, and significantly higher in coastal areas than those in inland areas.

**Table 1:** Characteristics of the studied population.

Characteristics	National	Rural area	Urban area	Coastal area	Inland area
	Mean±SD	Mean±SD	Mean±SD	Mean±SD	Mean±SD
Age (y)	26.7±4.3	25.1±3.8	28.0±4.2	28.2±4.5	26.1±4.1
BMI^1^	23.8±16.2	23.0±15.8	24.6±16.7	24.2±7.4	23.7±18.2
**Highest educational level of mothers**					
Primary school	18.4%	34.2%	4.3%	4.1%	23.1%
Junior middle school	37.4%	50.1%	26.0%	28.9%	40.2%
Senior high school	16.5%	9.5%	22.8%	24.4%	14.0%
College degree or above	27.6%	6.2%	47.0%	42.6%	22.7%
**Household income per capita (dollar /y)**					
<806.5	17.8%	31.3%	5.7%	1.4%	23.2%
806.5–1612.9	26.4%	42.1%	12.1%	5.3%	33.3%
1612.9–4838.7	42.8%	25.3%	58.6%	65.0%	5.6%
>4838.7	13.0%	1.3%	23.6%	28.4%	8.0%

^1^ BMI, body mass index, was calculated as body weight divided by height squared (kg/m^2^).

### The contents of B-vitamins in different stages of lactation

The contents of B-vitamins in different stages of lactation were shown in Table 2. The contents of six B-vitamins (thiamin, riboflavin, niacin, vitamin B-6, FAD and pantothenic acid) were all lowest in colostrum. The contents of thiamin (40.7 μg/L) and vitamin B-6 (80.7 μg/L) in 181–330 d mature milk were highest. The contents of riboflavin, FAD and pantothenic acid in transitional milk were highest (40.6 μg/L, 1162.8 μg/Land 2626.8 μg/L, respectively). There was no significant difference in the contents of riboflavin (33.6 μg/L vs 29.6 μg/L, *p* = 0.119) between 15–180 d mature milk and 181–330 d mature milk and they were lower than transitional milk (*p*<0.001). There was significant difference in the contents of niacin (687.0 μg/L vs 571.3 μg/L, *p*<0.001) and pantothenic acid (2213.0 μg/L vs 1895.5 μg/L, *p*<0.001) between 15–180 d mature milk and 181–330 d mature milk. There was no significant difference in the contents of niacin (661.3 μg/L vs 687.0 μg/L, *p* = 0.782) and pantothenic acid (2626.8 μg/L vs 2213.0 μg/L, *p* = 0.824) between transitional milk and 15–180 d mature milk. FAD content reached a plateau in mature milk after transitional period.

**Table 2:** The contents of B-vitamin in human milk among different stages of lactation (μg/L) ^1^ ^,^ ^2^ ^,^ ^3^.

Vitamins	0~7 d	8~14 d	15~180 d	181~330 d
	(n = 486)	(n = 416)	(n = 611)	(n = 232)
Thiamin	5.0 (2.4–8.8) ^bcd^	6.7 (3.3–12.6) ^acd^	21.1 (12.7–32.9) ^abd^	40.7 (28.5–60.7) ^abc^
	9.7±26.0	11.7±17.9	26.1±21.3	49.1±30.6
Riboflavin	29.3 (15.7–47.2) ^bc^	40.6 (26.7–65.8) ^acd^	33.6 (20.5–59.0) ^ab^	29.6 (16.1–57.0) ^b^
	46.3±73.2	60.5±83.0	51.3±63.2	45.4±49.7
Niacin ^4^	470.7 (281.6–776.0) ^bcd^	661.3 (407.2–1105.7) ^a^	687.0 (424.7–1065.7) ^ad^	571.3 (376.3–862.1) ^ac^
	639.3±590.3	860.5±689.6	887.9±782.3	667.5±440.8
Vitamin B-6 ^5^	4.6 (2.1–11.3) ^bcd^	16.1 (8.5–32.4) ^acd^	62.7 (40.9–93.9) ^abd^	80.7 (60.3–115.6) ^abc^
	16.1±69.8	26.4±31.8	72.7±53.0	91.3±45.3
FAD	808.7 (336.0–1499.4) ^bcd^	1162.8 (581.2–2009.6) ^a^	1023.9 (548.2–1637.8) ^a^	1057.2 (563.0–1620.7) ^a^
	1125.0±1122.9	1475.3±1229.1	1317.0±1196.1	1228.5±894.5
Pantothenicacid	1770.9 (780.9–2873.1) ^bcd^	2626.8 (1805.4–3762.7) ^ad^	2213.0 (1586.9–3348.1) ^ad^	1895.5 (1408.5–2740.2) ^abc^
	2199.1±2023.5	2880.6±1605.1	2672.0±1555.5	2203.6±1145.9

^1^ Data were expressed as the median (p25, p75) and mean ± SD.

^2^ Superscript a, b, c and d meant that significant difference compared with colostrums, transitional milk, 16–180 d mature milk and 181–330 d mature milk at *p*<0.05.

^3^ The analysis of variance (ANOVA) was conducted after the ln transformation.

^4^ Niacin = nicotinamide+nicotinic acid.

^5^ Vitamin B-6 = pyridoxal+ pyridoxine+pyridoxamine.

Percentages of B-vitamin contents in colostrums compared to transitional, 15–180 d mature milk and 181–330 d mature milk were shown in Table 3. These results were calculated using medians of Table 2 and meant that concentration of colostrums accounted for concentration of other stage milk. The contents of six B-vitamins were all lowest in colostrums, especially for vitamin B-6 for which the level was less than 10% that of mature milk. Thiamin was also much lower than transitional and mature milk.

**Table 3:** Percentage of B-vitamin contents in colostrum compared to transitional and mature milk (%).

Vitamins	Transitional milk	Mature milk	Mature milk
	(8~14 d)	(15~180 d)	(181~330 d)
Thiamin	74.6	23.7	12.3
Riboflavin	72.2	87.2	99.0
Niacin ^1^	71.2	68.5	82.4
Vitamin B-6 ^2^	28.6	7.3	5.7
FAD	69.5	79.0	76.5
Pantothenic acid	67.4	80.0	93.4

^1^ Niacin = nicotinamide+nicotinic acid.

^2^ Vitamin B-6 = pyridoxal+ pyridoxine+pyridoxamine.

### The contents of B-vitamins in rural and urban areas

The contents of B-vitamins in rural and urban areas were shown in Table 4. The contents of thiamin (colostrums and transitional milk), riboflavin (mature milk), niacin (each stage) and FAD (mature milk) were significantly lower in rural areas than in urban areas (*p*<0.05), however, the levels of vitamin B-6 (each stage) and pantothenic acid (colostrums and transitional milk) were significantly higher in rural areas than in urban areas (*p*<0.001). There was no difference in the contents of riboflavin (colostrums and transitional milk) and FAD (colostrums and transitional milk) between rural and urban areas.

**Table 4:** The contents of B-vitamin in human milk in rural and urban area (μg/L) ^1^ ^,^ ^2^ ^,^ ^3^.

Vitamins	Colostrum(n = 486)		Transitional milk(n = 416)		Mature milk(n = 841)	
	Rural area	Urban area	Rural area	Urban area	Rural area	Urban area
	(n = 200)	(n = 286)	(n = 182)	(n = 234)	(n = 460)	(n = 381)
Thiamin	3.2 ***	6.5 ***	4.8 ***	7.7 ***	26.4	25.6
	(1.2–5.3)	(3.8–10.5)	(1.6–10.7)	(5.0–14.2)	(15.2–46.6)	(15.2–37.6)
	5.1±7.3	12.8±33.0	9.1±12.8	13.8±20.7	34.6±29.5	29.8±21.8
Riboflavin	25.2	30.3	37.1	44.0	28.1 ***	38.1 ***
	(11.1–48.3)	(19.4–47.1)	(20.2–65.7)	(29.4–66.2)	(15.7–52.9)	(23.6–66.8)
	57.0±104.5	38.8±36.9	58.4±63.6	62.1±95.5	43.8±51.6	57.0±68.0
Niacin ^4^	314.0 ***	593.5 ***	577.8 ***	799.0 ***	540.8 ***	779.3 ***
	(213.5–467.4)	(403.6–968.4)	(360.6–881.6)	(467.5–1314.1)	(358.8–835.1)	(547.4–1195.2)
	386.2±270.7	807.3±675.2	680.4±474.6	1006.1±792.5	676.7±504.9	999.7±853.5
Vitamin B-6 ^5^	9.8 ***	3.5 ***	22.1 ***	13.3 ***	73.2 ***	60.8 ***
	(3.3–25.2)	(1.7–6.8)	(10.6–44.3)	(6.5–26.5)	(52.7–105.4)	(38.8–93.6)
	24.5±73.5	6.7±11.1	34.0±36.8	20.6±26.1	84.6±56.6	70.6±43.9
FAD	742.4	824.1	1261.9	1131.4	1005.3 *	1080.6 *
	(319.0–1489.4)	(357.5–1525.8)	(461.2–2052.8)	(631.0–1936.6)	(524.8–1571.5)	(594.2–1708.3)
	1026.9±969.0	1193.5±1215.9	1552.6±1357.0	1415.2±1119.0	1239.1±1081.0	1357.5±1168.0
Pantothenicacid	2163.6 ***	1448.5 ***	2957.2 ***	2439.6 ***	2062.5	2193.2
	(1113.7–3446.5)	(638.8–2538.1)	(2028.5–4054.8)	(1496.6–3437.5)	(1506.6–3055.6)	(1549.9–3321.4)
	2610.6±2180.8	1911.4±1856.1	3258.6±1727.7	2586.6±1439.3	2474.7±1372.1	2619.1±1576.0

^1^ Data were expressed as the median (p25, p75) and mean ± SD.

^2^ “*” meant significant difference between rural area and urban area at *p* <0.05, “***” meant significant difference between rural area and urban area at *p* <0.001.

^3^ The two independent sample *t*-test was conducted after the ln transformation.

^4^ Niacin = nicotinamide+nicotinic acid.

^5^ Vitamin B-6 = pyridoxal+ pyridoxine+pyridoxamine.

### The contents of B-vitamins in coastal and inland areas

The contents of B-vitamins in coastal and inland areas were shown in Table 5. The contents of thiamin (colostrums and transitional milk), riboflavin (transitional and mature milk) and niacin (each stage) were significantly higher in coastal areas than in inland areas (*p*<0.05) but the levels of vitamin B-6 (colostrums and mature milk) and FAD (colostrums and mature milk), and pantothenic acid (colostrums and transitional milk) were significantly lower in coastal areas than in inland areas (*p*<0.001).

**Table 5:** The contents of B-vitamin of human milk in coastal and areas (μg/L) ^1^ ^,^ ^2^ ^,^ ^3^.

Vitamins	Colostrum(n = 486)		Transitional milk(n = 416)		Mature milk(n = 843)	
	Coastal area	Inland area	Coastal area	Inland area	Coastal area	Inland area
	(n = 133)	(n = 353)	(n = 100)	)n = 316)	(n = 189)	(n = 654)
Thiamin	7.7 ***	4.2 ***	9.2 ***	6.1 ***	25.1	26.3
	(4.7–15.3)	(2.0–7.0)	(5.5–19.6)	(2.5–11.1)	(15.5–36.9)	(15.2–42.9)
	19.8±47.1	5.8±6.4	18.1±26.9	9.7±13.2	29.0±20.4	33.4±27.7
Riboflavin	31.9	28.2	43.9	39.9	42.6 ***	29.7 ***
	(20.4–49.5)	(13.8–46.9)	(27.4–72.9)	(25.5–63.0)	(26.6–73.9)	(17.6–54.4)
	37.1±25.6	49.7±84.2	72.2±134.2	56.7±58.0	64.0±81.4	45.6±51.3
Niacin ^4^	641.7 ***	381.0 ***	989.1 ***	589.2 ***	815.1 ***	583.4 ***
	(465.0–926.3)	(249.6–696.2)	(632.3–1472.6)	(365.2–952.9)	(612.9–1259.6)	(381.2–928.5)
	791.7±518.1	574.6±596.8	1183.2±847.2	762.4±600.2	1096.0±972.0	744.6±580.3
Vitamin B-6 ^5^	3.5 ***	6.4 ***	13.0 *	18.2 *	56.4 ***	71.2 ***
	(2.0–5.9)	(2.1–16.3)	(6.4–26.4)	(8.9–36.0)	(38.9–90.1)	(49.3–103.5)
	5.4±8.7	17.2±56.5	22.0±33.1	27.9±31.5	67.7±40.1	81.3±54.2
FAD	519.2 ***	923.1 ***	854.7	1229.5	831.1 ***	1088.7 ***
	(207.6–1180.3)	(390.7–1618.3)	(465.5–2227.6)	(676.0–1928.8)	(460.2–1257.5)	(595.4–1765.1)
	861.5±992.9	1224.2±1153.9	1301.8±1055.4	1530.3±1275.8	943.0±628.5	1393.7±1209.2
Pantothenicacid	944.5 ***	2070.6 ***	2184.4 ***	2801.6 ***	2121.0	2142.0
	(515.8–1935.7)	(1016.3–3231.5)	(1465.1–3177.5)	(1901.9–3866.3)	(1433.5–3107.4)	(1554.9–3205.5)
	1356.7±1179.6	2516.5±2179.6	2447.4±1525.4	3017.6±1607.6	2449.4±1498.2	2570.2±1460.1

^1^ Data were expressed as the median (p25, p75) and mean ± SD.

^2^ “*” meant significant difference between coastal area and inland area at *p* <0.05, “***” meant significant difference between coastal area and inland area at *p* <0.001.

^3^ The two independent sample *t*-test was conducted after the ln transformation.

^4^ Niacin = nicotinamide+nicotinic acid.

^5^ Vitamin B-6 = pyridoxal+ pyridoxine+pyridoxamine.

## Discussion

Our study analyzed the concentrations of six B-vitamins (thiamin, riboflavin and FAD, niacin, vitamin B-6 and pantothenic acid) in 1778 human milk samples from apparently healthy lactating women in China. The contents of all six B-vitamins were lowest in colostrums compared with transitional and mature milk. There was significant difference in contents of thiamin, riboflavin, niacin, vitamin B-6 and pantothenic acid between rural and urban areas and significant difference in contents of thiamin, riboflavin, niacin, vitamin B-6 and pantothenic acid between coastal and inland areas.

In this study, the contents of B-vitamins were lowest in colostrums, the ranges were consistent with some of the previous studies [13–16, 29, 36]. Ford *et al* [14] showed that the concentrations of thiamin, vitamin B-6, pantothenic acid and niacin increased with lactation stage increasing and the findings from Sakurai *et al* [15] study also supported such trend for thiamine, vitamin B-6 and riboflavin and the levels for some vitamins such as niacin and pantothenic acid remained almost constant during lactation period. However, the relatively lower B-vitamin contents in colostrum and their significance to neonates remain unclear. An animal study [38] has found that feeding colostrum had little impacts on offspring’s plasma vitamin B-6 status. Vitamin B-6 status of offspring was higher at birth and decreased on day 2 after birth regardless of feeding colostrums or water. This may suggest that newborns might have some vitamin B-6 store during pregnancy. With lactation stage increasing, the concentrations of thiamin and vitamin B-6 increased, the contents of niacin and pantothenic acid remained nearly unchanged, which was similar to the trend of Sakurai *et al* [15]. The contents of riboflavin reached a peak in transitional milk and the contents of FAD were similar in transitional milk and mature milk. Our results were supported by some references [13, 15, 16, 20, 29, 39]. However, the results were lower than those reported in other studies [14, 30, 31].

Generally, such difference could be reflected the difference in the milk sampling collection, analytical methods, loss during storage and pretreatment as well as race and maternal dietary intakes. The contents of B-vitamins in human milk are usually determined by using HPLC or UPLC-MS/MS method and microbiological assays. A comparison of HPLC, UPLC-MS/MS and microbiological assays shows relatively wide ranges for B-vitamin contents in human milk (Table 6) [13–16]. LC methods in present study only analyze a specific form of these vitamins (i.e. free form) so that the contents of these vitamins (thiamin, riboflavin, niacin and vitamin B-6) analyzed by LC methods were much lower than the microbiological assays, because free forms of these vitamins (thiamin, riboflavin, nicotinamide and vitamin B-6) were only part of the total vitamin contents [14, 40]. By contrast, LC methods gave similar results as those microbiological assays for those vitamins (e.g. pantothenic acid) because free pantothenic acid was the major form in human milk [18]. In this study the free forms of B-vitamins were analyzed and the total amount for those B-vitamins can be estimated based on the ratios reported from previous studies. Specially, our analysis was good in accordance with the results from Asian studies in Inner Mongolia and in Japan [13, 15, 39]. Therefore, the level of B-vitamins in human milk may be affected by the different life style and diet regime [13]. Moreover, the methods sampling breast milk can result in obvious variation in the contents of B-vitamins in human milk [13, 15, 16, 20, 29, 39]. Two separate 5 or 10 mL milk samples at the beginning and the end of the feeding were collected in some researches [18, 24, 26, 29]. Their results indicated that the contents of B-vitamins were significantly different between fore milk and hind milk. Therefore, the B-vitamin levels in a spot sample (5 or 10 mL) could not reflect the actual contents [25, 28, 31]. Human milk composition from the same mother may vary over the course of the day [36] and the analyzed method, the different population and sample collection could affect the levels of B-vitamins in human milk. To reduce the impacts of sampling on nutrient contents, milk samples collection was standardized in present study. McCullough *et al* found that the Egyptian infants can experience a convulsive seizure if the vitamin B-6 concentration in human milk was lower than 46.3 μg/L [40]. Some of our results were similar to this restricted data. However, only free forms of vitamin B-6 were analyzed and the bound forms, e.g. pyridoxal-5’-phosphate, were not considered in this study.

**Table 6:** The comparison of UPLC-MS/MS, HPLC and Microbiological assays for B-vitamins in human milk (μg/L).

Vitamins	UPLC-MS/MS [16]	HPLC [13, 15]	Microbiological assays [14]
Thiamine	2~221	9~199	13~360
Riboflavin	0~845	70–242	120~440
Niacin	2~3179	292~3240	260~2800
Vitamin B-6	6~692	14~120	2.3~180
Pantothenic acid		1400~3390	480~3700

In general, the contents of thiamin, riboflavin and FAD, and niacin in breast milk were lower in rural areas than in urban areas. Previous studies also found such difference [22, 23]. The levels of these B-vitamins in human milk were closely related to maternal dietary intake or nutritional status [27–31]. Dietary intakes of thiamin, niacin and riboflavin were shown to be lower for lactating women in rural areas of China [22, 33, 41–45]. Additionally, with consuming more and more refined cereals, the intakes of thiamin and riboflavin from these products tends to decrease [37]. In generally, healthy diet habits were closely related to higher education level, occupational situation and economic status [46, 47]. Stelmach *et al* [48] suggested that the higher education may be a strong and consistent predictor of good health. According to the characteristics of the study population, levels of education and income were higher in urban area than rural area, which should be related to the content difference of thiamin, riboflavin and FAD between rural and urban areas.

The contents of niacin in human milk were higher in coastal area than in inland area. Niacin widely exists in fishes and seafood, such as mussel, crab meat, small shrimp, mackerel, mandarin fish and so on [49]. The contents of riboflavin were higher in coastal area than in inland area after 7 days of postpartum. Some fishes contain abundant riboflavin, such as finless eel slice and babylon shell [49]. The lactating women in coastal area had more possibility to eat these marine products, which may contribute to the content difference of niacin and riboflavin between coastal and inland areas. The levels of education and income were higher in coastal area than inland area, which may be related to the differences of contents of thiamin, riboflavin and FAD between coastal area and inland area. However, the levels of vitamin B-6 and pantothenic acid were contrary to the social-economic status. The possible reason was that pantothenic acid widely exists in various foods [37].

In fact, we have analyzed some minor vitamins in human milk such as nicotinic acid, pyridoxine and pyridoxamine. However, the contents of them were too low to be detected in part of samples so that we did not report these values individually in present paper. We gave the sum of pyridoxal, pyridoxine and pyridoxamine as the vitamin B-6 and the sum of nicotinamide and nicotinic acid as niacin. In our study, 4.3% mothers (n = 76) had taken nutrient supplements during lactatione. The major supplements included calcium tablet, protein, cubilose, and so on. Only five mothers received the vitamin supplements and they were excluded already.

The limitation of the study was that the free forms of B-vitamins only were analyzed so that the findings can not reflect the total level for these vitamins including bound forms. For determination of bound forms of B-vitamins, e.g. thiamin monophosphate, pyridoxal-5’-phosphate, it should use more intensive pretreatment such as enzymolysis and acidolysis and different enzyme or different treatment condition for specific vitamin which would be a limitation for simultaneous analysis of free and bound vitamin forms. In subsequent analysis, we should consider how to analyze the bound forms of B-vitamins individually. Moreover, this study was a cross-sectional survey, which limits the possibility to study the changes of vitamin levels from the same subjects over the course of lactation as a longitudinal study would do.

In summary, the contents of six B-vitamins (thiamin, riboflavin, FAD, vitamin B-6, niacin and pantothenic acid) were lower in colostrums than in transitional and mature milk in China. The levels of B-vitamins were consistent with previous reports, but larger variations were present which could reflect the differences in breast milk collection measures and different analytical methods. Our findings can reflect the general level of these B-vitamins in human milk from apparently healthy lactating women in China. The contents of riboflavin, FAD, vitamin B-6 and pantothenic acid were affected by lactation stage, environment and region.

## Supporting Information

S1 Table The original data of analysis for samples. (XLSX)

S1 Fig The chromatograms of target components. (DOC)
