# Supplementary material for: B-Vitamin Levels in Human Milk among Different Lactation Stages and Areas in China
Source: PLoS One. 2015 Jul 17;10(7):e0133285. doi: 10.1371/journal.pone.0133285 (PMC4505892; doi:10.1371/journal.pone.0133285)
Supplement: S1 Fig — (DOC) [file pone.0133285.s002.doc]

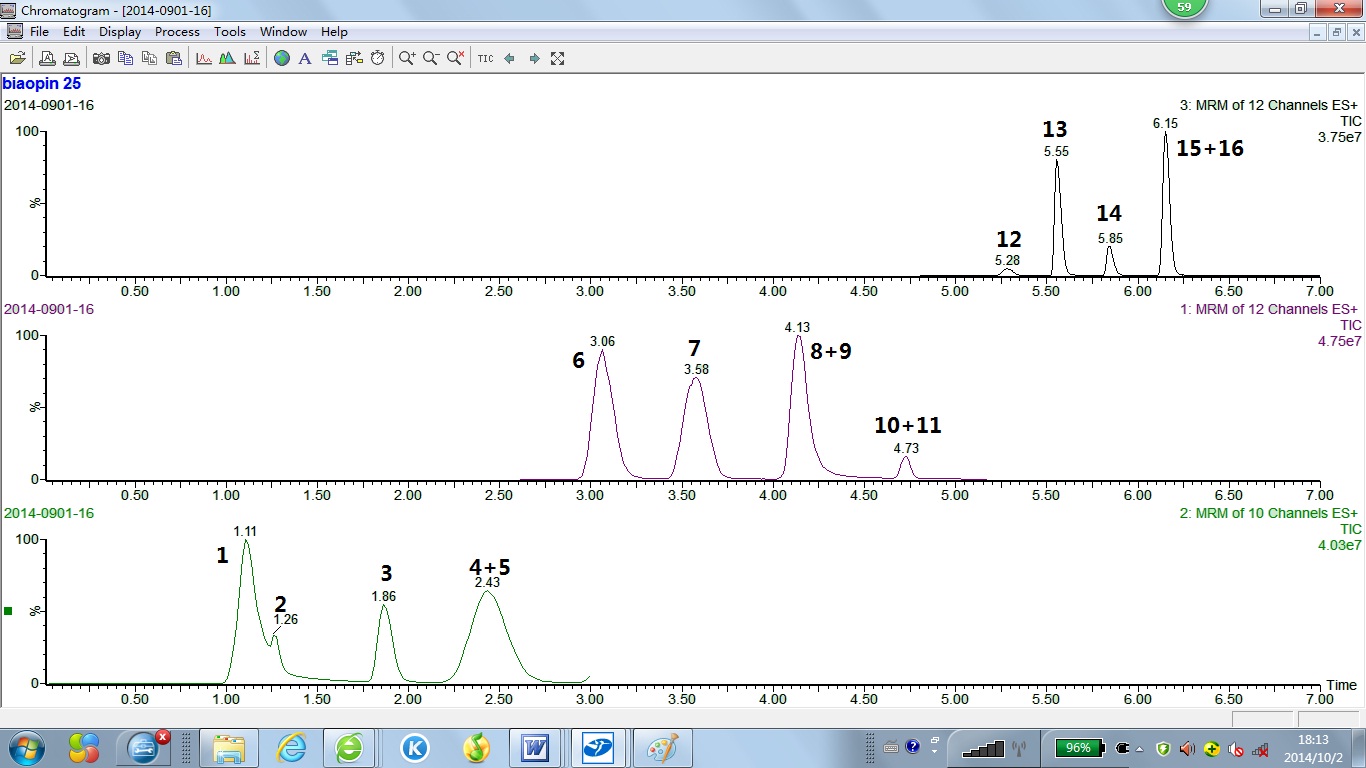


(A)


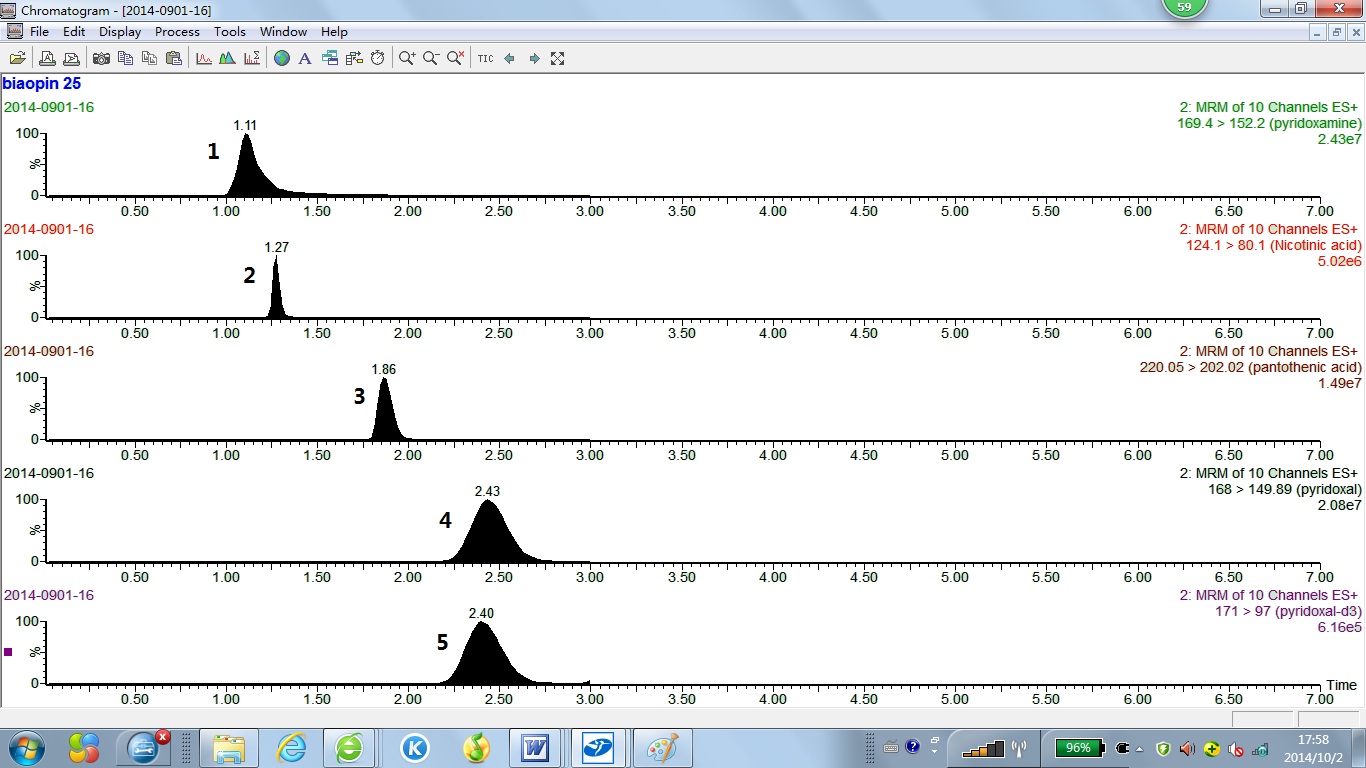


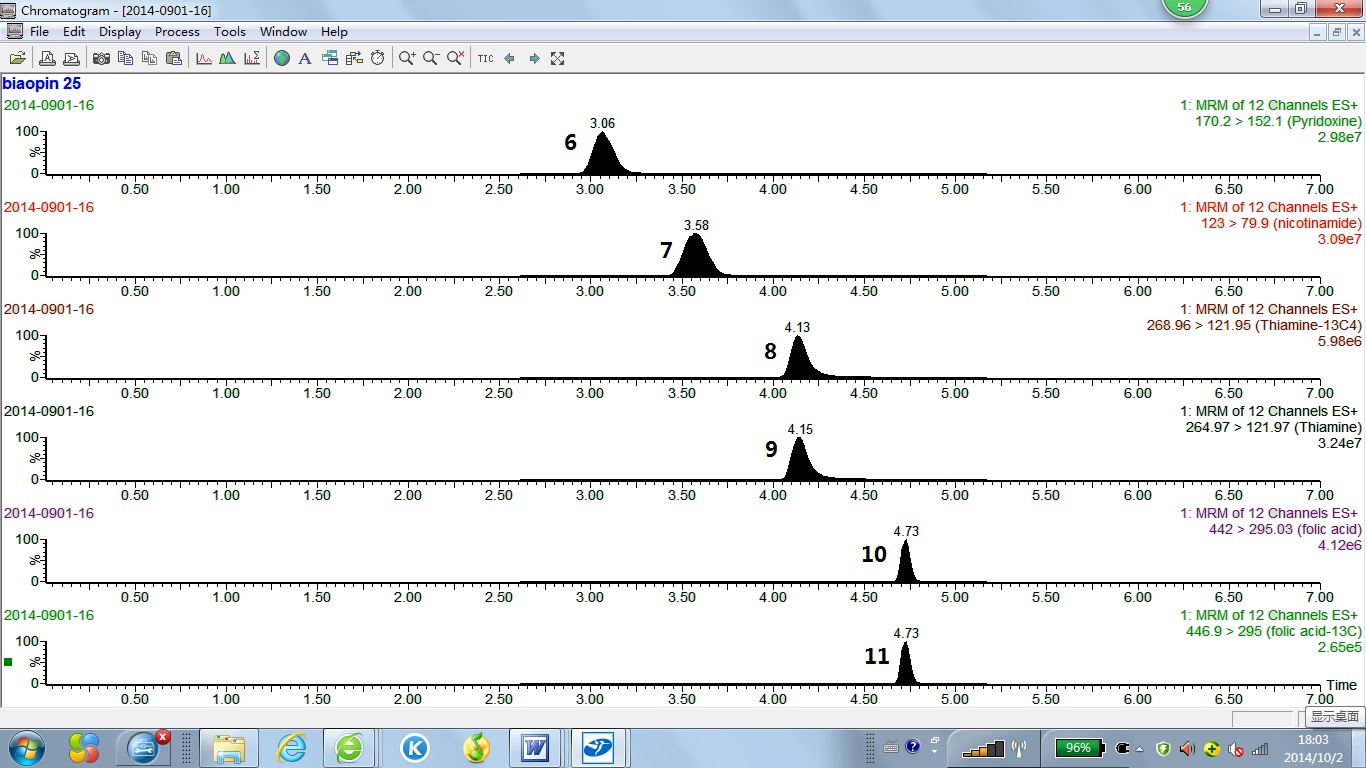


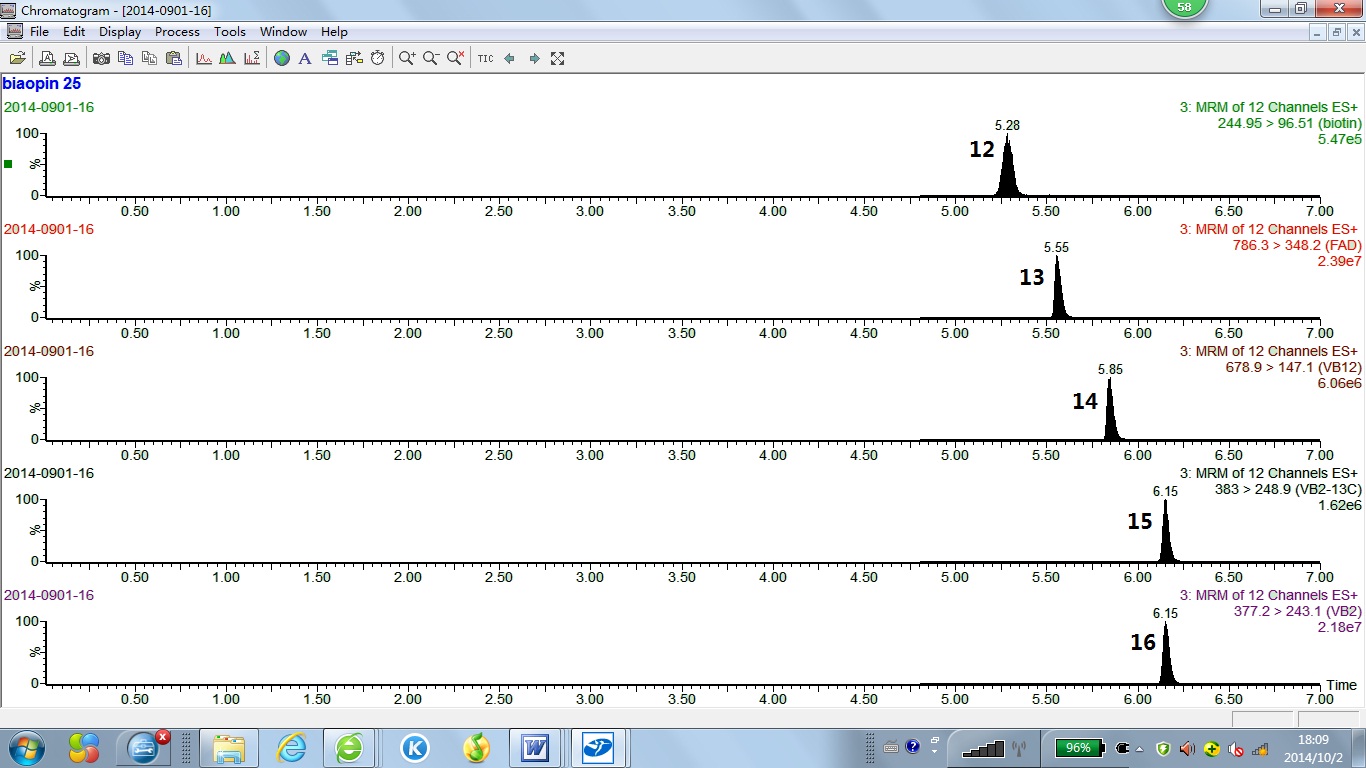


(B)

Fig. 1. (A) The total ion chromatograms TIC and (B) quantitative daughter ion chromatograms of 16 target components: (1) pyridoxamine; (2) nicotinic acid; (3) pantothenic acid; (4) pyridoxal; (5) pyridoxal-d3; (6) pyridoxine; (7) nicotinamide; (8) thiamin-13C3; (9) thiamin; (10) folic acid ; (11) folic acid-13C3; (12) biotin; (13) FAD; (14) cobalamin; (15) riboflavin- 13C4, 15N2; (16) riboflavin.
